# Supplementary material for: LTA4H extensively associates with mRNAs and lncRNAs indicative of its novel regulatory targets
Source: PeerJ. 2023 Mar 10;11:e14875. doi: 10.7717/peerj.14875 (PMC10010175; doi:10.7717/peerj.14875)
Supplement: File S2 [file peerj-11-14875-s005.zip › Raw data files/Motif_results/piranha/LTA4H_IP_2_vs_LTA4H_input_2/homerResults.html]

.// - Homer de novo Motif Results


# Homer *de novo* Motif Results (.//)

Known Motif Enrichment Results  
Gene Ontology Enrichment Results  
If Homer is having trouble matching a motif to a known motif, try copy/pasting the matrix file into
STAMP  
More information on motif finding results: HOMER
| Description of Results
| Tips
  
Total target sequences = 9616  
Total background sequences = 79743  
\* - possible false positive  

|  |  |  |  |  |  |  |  |  |
| --- | --- | --- | --- | --- | --- | --- | --- | --- |
| Rank | Motif | P-value | log P-pvalue | % of Targets | % of Background | STD(Bg STD) | Best Match/Details | Motif File |
| 1 |  | 1e-220 | -5.089e+02 | 14.14% | 4.92% | 21.6bp (23.5bp) | hsa-miR-555 MIMAT0003219 Homo sapiens miR-555 Targets (miRBase)(0.716) More Information \| Similar Motifs Found | motif file (matrix) |
| 2 |  | 1e-85 | -1.968e+02 | 26.27% | 17.68% | 18.2bp (29.9bp) | hsa-miR-4268 MIMAT0016896 Homo sapiens miR-4268 Targets (miRBase)(0.685) More Information \| Similar Motifs Found | motif file (matrix) |
| 3 |  | 1e-62 | -1.432e+02 | 12.37% | 7.21% | 15.7bp (22.3bp) | hsa-miR-875-3p MIMAT0004923 Homo sapiens miR-875-3p Targets (miRBase)(0.652) More Information \| Similar Motifs Found | motif file (matrix) |
| 4 |  | 1e-48 | -1.111e+02 | 8.86% | 5.01% | 17.8bp (27.7bp) | hsa-miR-3135b MIMAT0018985 Homo sapiens miR-3135b Targets (miRBase)(0.702) More Information \| Similar Motifs Found | motif file (matrix) |
| 5 |  | 1e-38 | -8.979e+01 | 3.32% | 1.34% | 26.9bp (33.5bp) | hsa-miR-574-5p MIMAT0004795 Homo sapiens miR-574-5p Targets (miRBase)(0.724) More Information \| Similar Motifs Found | motif file (matrix) |
| 6 |  | 1e-35 | -8.187e+01 | 7.21% | 4.20% | 19.8bp (26.7bp) | hsa-miR-425\* MIMAT0001343 Homo sapiens miR-425\* Targets (miRBase)(0.685) More Information \| Similar Motifs Found | motif file (matrix) |
| 7 |  | 1e-32 | -7.471e+01 | 0.81% | 0.10% | 26.5bp (17.3bp) | hsa-miR-323-3p MIMAT0000755 Homo sapiens miR-323-3p Targets (miRBase)(0.685) More Information \| Similar Motifs Found | motif file (matrix) |
| 8 |  | 1e-31 | -7.357e+01 | 16.30% | 11.93% | 15.3bp (19.8bp) | hsa-miR-4496 MIMAT0019031 Homo sapiens miR-4496 Targets (miRBase)(0.719) More Information \| Similar Motifs Found | motif file (matrix) |
| 9 |  | 1e-30 | -7.053e+01 | 9.75% | 6.43% | 19.2bp (20.7bp) | hsa-miR-4472 MIMAT0018999 Homo sapiens miR-4472 Targets (miRBase)(0.669) More Information \| Similar Motifs Found | motif file (matrix) |
| 10 |  | 1e-28 | -6.567e+01 | 8.95% | 5.88% | 17.8bp (22.4bp) | hsa-miR-509-5p MIMAT0004779 Homo sapiens miR-509-5p Targets (miRBase)(0.657) More Information \| Similar Motifs Found | motif file (matrix) |
| 11 |  | 1e-28 | -6.539e+01 | 3.31% | 1.56% | 25.1bp (30.7bp) | hsa-miR-126 MIMAT0000445 Homo sapiens miR-126 Targets (miRBase)(0.625) More Information \| Similar Motifs Found | motif file (matrix) |
| 12 |  | 1e-26 | -6.187e+01 | 2.42% | 1.01% | 18.7bp (26.3bp) | hsa-miR-141\* MIMAT0004598 Homo sapiens miR-141\* Targets (miRBase)(0.722) More Information \| Similar Motifs Found | motif file (matrix) |
| 13 |  | 1e-25 | -5.892e+01 | 21.76% | 17.30% | 19.6bp (23.7bp) | hsa-miR-4530 MIMAT0019069 Homo sapiens miR-4530 Targets (miRBase)(0.686) More Information \| Similar Motifs Found | motif file (matrix) |
| 14 |  | 1e-23 | -5.314e+01 | 2.43% | 1.10% | 20.0bp (24.8bp) | hsa-miR-4792 MIMAT0019964 Homo sapiens miR-4792 Targets (miRBase)(0.670) More Information \| Similar Motifs Found | motif file (matrix) |
| 15 |  | 1e-22 | -5.232e+01 | 4.17% | 2.35% | 30.2bp (24.1bp) | hsa-miR-4513 MIMAT0019050 Homo sapiens miR-4513 Targets (miRBase)(0.658) More Information \| Similar Motifs Found | motif file (matrix) |
| 16 |  | 1e-21 | -5.052e+01 | 10.58% | 7.62% | 20.8bp (23.1bp) | hsa-miR-136\* MIMAT0004606 Homo sapiens miR-136\* Targets (miRBase)(0.675) More Information \| Similar Motifs Found | motif file (matrix) |
| 17 |  | 1e-17 | -4.072e+01 | 24.34% | 20.47% | 16.2bp (19.7bp) | hsa-miR-4534 MIMAT0019073 Homo sapiens miR-4534 Targets (miRBase)(0.660) More Information \| Similar Motifs Found | motif file (matrix) |
| 18 |  | 1e-17 | -3.995e+01 | 0.43% | 0.06% | 19.6bp (21.0bp) | hsa-miR-885-3p MIMAT0004948 Homo sapiens miR-885-3p Targets (miRBase)(0.631) More Information \| Similar Motifs Found | motif file (matrix) |
| 19 |  | 1e-16 | -3.870e+01 | 6.36% | 4.36% | 18.2bp (18.6bp) | hsa-miR-495 MIMAT0002817 Homo sapiens miR-495 Targets (miRBase)(0.698) More Information \| Similar Motifs Found | motif file (matrix) |
| 20 |  | 1e-14 | -3.399e+01 | 0.88% | 0.29% | 23.3bp (19.5bp) | hsa-miR-3619-5p MIMAT0017999 Homo sapiens miR-3619-5p Targets (miRBase)(0.847) More Information \| Similar Motifs Found | motif file (matrix) |
| 21 |  | 1e-13 | -3.137e+01 | 0.26% | 0.02% | 21.8bp (8.3bp) | hsa-miR-2117 MIMAT0011162 Homo sapiens miR-2117 Targets (miRBase)(0.628) More Information \| Similar Motifs Found | motif file (matrix) |
| 22 |  | 1e-13 | -3.098e+01 | 0.55% | 0.14% | 12.0bp (25.6bp) | hsa-miR-4330 MIMAT0016924 Homo sapiens miR-4330 Targets (miRBase)(0.736) More Information \| Similar Motifs Found | motif file (matrix) |
| 23 \* |  | 1e-11 | -2.699e+01 | 23.21% | 20.14% | 19.8bp (24.6bp) | hsa-miR-3115 MIMAT0014977 Homo sapiens miR-3115 Targets (miRBase)(0.655) More Information \| Similar Motifs Found | motif file (matrix) |
